# Genetics of Premature Ovarian Insufficiency: From Molecular Architecture to Precision Medicine

**DOI:** 10.3390/ijms27198662

**Published:** 2026-09-28

**Authors:** Vanessa Sousa, Gabriel Livera, Micheline Misrahi

**Affiliations:** 1 Cytogenetics Laboratory, School of Medicine and Biomedical Sciences, University of Porto, Rua Jorge Viterbo Ferreira 228, 4050-313 Porto, Portugal; 2 Unit for Multidisciplinary Research in Biomedicine, School of Medicine and Biomedical Sciences, University of Porto, Rua Jorge Viterbo Ferreira 228, 4050-313 Porto, Portugal; 3 Stabilité Génétique Cellules Souches et Radiations, LDG/iRCM/IBFJ, Inserm, CEA, Université Paris Cité, F-92260 Fontenay-Aux-Roses, France; 4 Stabilité Génétique Cellules Souches et Radiations, LDG/iRCM/IBFJ, Inserm, CEA, Université Paris-Saclay, F-92260 Fontenay-Aux-Roses, France; 5 Unité de Génétique Moléculaire des Maladies Métaboliques et de la Reproduction, Hôpital Bicêtre AP-HP, Hôpitaux Universitaires Paris-Saclay, Faculté de Médecine, Université Paris-Saclay, 94275 Le Kremlin-Bicetre, France

**Keywords:** premature ovarian insufficiency, female infertility, genetics, meiosis, DNA repair, follicular growth, mitochondrial function, molecular pathophysiology, targeted therapies, precision medicine

## Abstract

Premature ovarian insufficiency (POI) affects 1–3.7% of women and causes infertility and long-term morbidity. Next-generation sequencing has identified more than 130 POI-associated genes. The diagnostic yield of genetic testing depends on cohort size and the quality and scope of the genetic analysis, reaching 29.3% in our largest international cohort reported to date. Major biological pathways involve meiosis/DNA repair, follicular growth, and mitochondrial/metabolic functions. Meiosis/DNA-repair defects can impair fetal establishment of the ovarian reserve and, depending on the gene, variant, and allelic state, may be associated with chromosomal instability or cancer predisposition. This could enable early identification of selected at-risk patients, oncogenetic assessment, and cancer-prevention strategies. In contrast, alterations affecting follicular growth, metabolism, or mitochondrial function may be associated with persistence of a residual follicular pool, revealing a limited window of opportunity for timely fertility preservation in selected patients and at-risk relatives. Genetic diagnosis provides actionable information for etiological classification, and can reveal a syndromic condition, guide family screening, and, in selected genotypes, inform comorbidity assessment and early fertility preservation. It may also guide future mechanism-based therapies. By translating molecular findings into individualized reproductive and long-term care, POI provides a paradigmatic model of precision medicine in female infertility and reproductive endocrinology.

## 1. Introduction

Premature ovarian insufficiency (POI) affects approximately 1–3.7% of women before age 40, with prevalence estimates varying according to the population studied [1,2]. It is diagnosed by amenorrhea or oligomenorrhea over 4 months, with a plasma FSH level ≥ 25 IU/L [2]. POI is a major cause of infertility. However, ovarian activity may intermittently resume, and spontaneous pregnancies can occur, indicating that ovarian insufficiency is not invariably permanent [1].

POI has multiple causes, including iatrogenic factors (chemotherapy, surgery, radiotherapy), infections and autoimmune disorders. However, ~70% of cases remain unexplained [1].

A genetic contribution to POI is supported by twin studies and familial aggregation. A positive family history is reported in 4–31% of cases, and first-degree female relatives of affected women have a markedly increased risk, with an odds ratio of 4.6 (95% CI, 3.3–6.5) [1,2,3]. Heritability estimates for age at natural menopause range from approximately 44% to 85%, depending on the population and study design [4,5,6].

POI may occur as an isolated disorder or as part of a multisystem syndrome. It is associated with substantial morbidity related to sex steroid deficiency, including osteoporosis, cardiovascular disease, and increased incidence of Alzheimer disease and other dementias [1,2]. Remarkably, epidemiological studies have shown that early menopause is independently associated with reduced life expectancy and increased all-cause mortality, particularly in women with POI [7,8,9,10,11].

Genetic studies have provided a mechanistic explanation for this association by highlighting the major role of meiosis and DNA repair genes in physiological menopause and POI [1,12]. Indeed, these genes are also implicated in cancer predisposition and longevity.

This review synthesizes previously published work from our team, including genetic analyses conducted in a large international cohort of patients with POI and informative families. It highlights the resulting insights into POI genetic architecture, genotype–phenotype correlations, and the translational implications for diagnosis, fertility prognosis, associated comorbidities, and emerging targeted therapeutic strategies. Particular emphasis is placed on mechanisms involving meiosis and DNA repair, follicular growth, and metabolic or mitochondrial pathways, which together account for most genetically characterized forms of POI. We also provide an updated and curated list of genes implicated in POI, organized by major biological pathways. Together, these advances support genetically guided clinical management and precision medicine in POI.

## 2. Molecular Architecture and Translational Genetics of POI

### 2.1. A Leap Forward in the Identification of Genetic Causes of POI

Chromosomal abnormalities are identified in approximately 10–13% of patients, mainly involving the X chromosome [2]. When the karyotype is normal, the search for monogenic causes is performed. Until recently, the only gene analysis performed in most centers was the search for premutation of the *FMR1* gene found in 3–5% of patients with sporadic POI [13].

The advent of next-generation sequencing (NGS), including whole-exome sequencing (WES), has significantly accelerated the discovery of genes involved in POI [1]. These technologies enable analysis of the coding regions of approximately 20,000 genes or, through whole-genome sequencing (WGS), of the complete genome sequence. More than 130 genes have now been implicated in POI, highlighting its remarkable genetic heterogeneity [1,14,15,16,17,18,19,20,21,22,23,24,25,26,27,28,29,30,31,32,33,34,35,36,37,38,39,40,41,42,43,44,45]. Table S1 provides a curated catalog of genes implicated in POI, classified according to molecular pathway, mode of inheritance, associated phenotype, and involvement in male infertility. The catalog was based on our previously established 2022 list [14] and updated through a comprehensive review of the additional literature published over the past five years. Only genes supported by evidence in patients with POI were retained, with priority given to genes reported in several independent families and/or supported by functional studies.

### 2.2. Genetic Landscape of POI in a Large International Cohort

Large cohorts are required to assess the feasibility of routine genetic diagnosis in POI and to define the contribution of individual genes, particularly in sporadic cases.

In a previously published study, we evaluated first-line genetic diagnosis in a large international cohort of 375 patients with POI, predominantly of Caucasian origin, including 70 families (Figure 1) [14]. All patients had unexplained POI, a normal karyotype, and normal *FMR1* testing. A custom-made NGS-POI panel comprising 88 validated POI genes was developed. For clinical diagnosis, only genes with established evidence of causality for POI were considered, and variant interpretation followed the criteria of the American College of Medical Genetics and Genomics (ACMG) [46].

This study established a molecular diagnosis in 29.3% of cases [14] (Figure 1). Diagnostic yield was higher in familial or consanguineous POI, reaching 36.7% in North African and Turkish patients, and was particularly high in syndromic POI (58.3%) [14].

Combined with parallel *FMR1* testing, using a specific assay, this strategy enables a genetic diagnosis in one-third of patients with unexplained POI, representing a major advance in the field.

This high diagnostic yield supports the use of genetic testing in routine clinical practice for all unexplained POI.

### 2.3. Mechanistic Insights into POI: Translational Implications for Clinical Stratification

Our previous study [14] showed that molecular classification of POI provides a framework for clinically meaningful patient stratification. Despite marked genetic heterogeneity, three major molecular pathway groups accounted for more than 90% of identified genetic causes: meiosis and DNA repair (37.4%), follicular growth (35.4%), and metabolism and mitochondrial function (19%) (Figure 1) [14].

Additional genes are involved in ovarian development, inflammation and immune regulation, transcriptional and translational regulation, mitochondrial autophagy, and follicular atresia.

It should be noted that, in ~8.5% of cases, POI represents the initial or sole manifestation of a broader multisystemic genetic disorder, highlighting the need for a comprehensive clinical evaluation in all patients with POI.

A subsequent independent WES study of 1030 unrelated Chinese patients with POI provided an important comparison with our international cohort [16]. The two studies differed in population and design. Our cohort included 375 women with unexplained POI from several geographic origins, predominantly European, including 70 familial cases; patients had normal karyotype and *FMR1* testing before monogenic analysis and were investigated using either a custom 88-gene NGS panel or WES [14]. In contrast, Ke et al. studied 1030 unrelated Chinese patients with idiopathic POI, after exclusion of chromosomal abnormalities and recognized non-genetic causes, and performed WES in all patients [16]. Variant interpretation in our cohort was based on ACMG criteria, with molecular diagnosis restricted to pathogenic (P) and likely pathogenic (LP) variants. In the Chinese study, variants in established POI genes were interpreted using ACMG/ClinVar criteria. In parallel, a case–control burden analysis tested whether rare loss-of-function variants were enriched in specific genes in patients with POI compared with 5000 controls, thereby identifying additional candidate genes. The diagnostic endpoints are therefore not identical. In our cohort, P/LP variants established a molecular diagnosis in 110/375 patients (29.3%). In the Chinese cohort, P/LP variants in established POI genes were identified in 193/1030 patients (18.7%). When the additional candidate genes identified by the burden analysis were also considered, the estimated overall genetic contribution increased to 242/1030 patients (23.5%). Despite these differences in ancestry, patient selection, sequencing strategy, and analytical approach, the two cohorts show substantial convergence at the pathway level.

Meiosis and DNA-repair/homologous-recombination genes represented the largest molecular category in both studies, accounting for 37.4% in our cohort and 48.7% in the Chinese cohort. In our cohort, follicular-growth genes accounted for 35.4% and mitochondrial/metabolic genes for 19% [14]. In the Chinese cohort, mitochondrial, metabolic, and autoimmune genes collectively accounted for 22.3% of genetically characterized cases, while the burden analysis additionally identified genes involved in gonadogenesis, folliculogenesis, and oocyte maturation or ovulation [16]. Thus, the strongest reproducible finding across the two populations is the major contribution of meiosis/DNA repair, with additional support for mitochondrial/metabolic and follicular-development mechanisms.

In addition, Gorsi et al. analyzed 291 women by WES and independently identified enrichment of functional categories including DNA damage and repair and meiosis/cell division, although the study was designed primarily for gene discovery and pathway enrichment rather than ACMG-based diagnostic classification [47].

Other published sequencing series are less directly comparable because of small cohort size, analysis restricted to a limited number of POI-related genes, or differences in variant interpretation, including the incorporation of variants of uncertain significance or candidate-gene variants among reported genetic findings rather than restricting positive results to P/LP variants according to ACMG criteria. These methodological differences limit direct quantitative comparison of diagnostic yields across studies [2].

A major and unexpected finding from genetic studies is that the predominant molecular pathway implicated in POI comprises genes involved in meiosis and DNA repair. These genes are essential for the establishment of ovarian reserve during fetal life, and their alteration leads to impaired meiotic progression and early follicular depletion [1,14].

Importantly, many meiosis and DNA repair genes are also involved in cancer susceptibility and longevity, highlighting shared molecular mechanisms linking reproductive lifespan, infertility, genome stability, tumor susceptibility, and aging [1,14,48].

### 2.4. Molecular Diagnosis as the Basis for Clinical Stratification

Molecular diagnosis links the causal pathway to clinically relevant outcomes. Defects in meiosis and DNA repair generally indicate impaired establishment or accelerated depletion of the ovarian reserve and may reveal chromosomal instability or tumor susceptibility. By contrast, defects affecting follicular growth, metabolic, mitochondrial, immune, or developmental pathways may be associated with a residual follicular pool and with distinct extra-ovarian manifestations. Thus, the causal gene and its underlying molecular mechanism, rather than the POI phenotype alone, enable patient stratification and inform clinical surveillance and multidisciplinary care.

The principal clinical consequences of molecular diagnosis include etiological clarification, recognition of syndromic or cancer-predisposition phenotypes, assessment of the residual ovarian reserve, individualized fertility counseling and preservation, and cascade testing of relatives. These implications are discussed below within each molecular pathway to maintain a direct link between clinical management and its biological basis. However, whereas the recurrent involvement of major biological pathways is supported by independent cohorts, genotype–phenotype and clinical observations for individual rare variants are frequently based on small patient series or individual families and should therefore be interpreted as gene-specific evidence rather than as population-level estimates.

### 2.5. Implementation of Molecular Testing in Clinical Practice

Translation into clinical practice requires pre-test information to the patient and signed informed consent, followed by a stepwise testing strategy beginning with karyotype analysis. In patients with a normal karyotype, *FMR1* testing and NGS should then be performed in parallel, with standardized variant interpretation based on ACMG guidelines. Molecular findings should subsequently be integrated with the clinical phenotype, genotype–phenotype correlations, and familial segregation studies. Post-test counseling should address reproductive prognosis, associated comorbidities, implications for relatives, and gene-specific follow-up. The selected examples below illustrate how this molecular framework can inform and modify clinical management.

The optimal NGS strategy depends on the clinical context and available resources. Targeted panels provide high and uniform coverage of established POI genes, whereas WES is particularly useful in syndromic cases, consanguineous families, or after negative first-line testing and allows periodic reanalysis. WGS may additionally detect structural and non-coding variants but is not yet routinely available. Copy-number variant analysis should be included when technically feasible. Negative results should be periodically reconsidered as new POI genes are identified and validated.

### 2.6. Meiosis and DNA Repair Genes in POI: Links to Cancer Susceptibility

During fetal life, hundreds of programmed DNA double-strand breaks (DSBs) are generated simultaneously during meiotic prophase I and must be accurately repaired to ensure homologous recombination (HR). Postnatally, DNA damage accumulates in oocytes as a result of metabolic activity, oxidative stress, aging, and environmental exposures [1]. Failure to repair this damage activates DNA damage checkpoints, leading to oocyte apoptosis and progressive depletion of the ovarian reserve.

#### 2.6.1. Fanconi Pathway Genes Can Cause Isolated POI Without Fanconi Anemia or Cancer

The Fanconi anemia (FA) pathway is a major DNA repair pathway that acts in close cooperation with homologous recombination to maintain genome stability [48]. Patients with biallelic mutations in this pathway typically develop FA, a rare and severe autosomal recessive disorder characterized by bone marrow failure, congenital anomalies, marked chromosomal instability, and predisposition to acute myeloid leukemia and other cancers. This chromosomal instability is exacerbated by mitomycin C (MMC), a DNA interstrand cross-linking agent that arrests replication forks [48].

Our work provided the first evidence that biallelic defects in key DNA repair genes of the Fanconi anemia pathway, including *FANCM*, *BRCA2*, and *BRCA1*, can cause isolated POI [17,18,31]. The reported patients initially presented without clinical or hematological signs of Fanconi anemia and without a personal or family history of cancer. Nevertheless, cytogenetic studies revealed increased chromosomal fragility under genotoxic stress induced by MMC. Residual Fanconi pathway activity appeared sufficient to maintain genomic stability in somatic cells under basal conditions but insufficient during fetal meiosis, when hundreds of programmed DNA double-strand breaks occur simultaneously within each oocyte.

These findings demonstrate that partial impairment of DNA-repair pathways can cause ovarian dysfunction before overt somatic manifestations become apparent.

Our previously published work further extended this observation to SPIDR, a protein interacting with RAD51 and BLM at the interface of homologous recombination and genome-stability pathways. In a patient with isolated POI carrying a homozygous nonsense *SPIDR* variant, we demonstrated a marked increase in MMC-induced chromosome breaks and aberrant metaphases, consistent with impairment of the RAD51-mediated DNA-repair pathway [41]. These findings provided the first evidence that *SPIDR* deficiency causes chromosomal instability in humans and established its causal role in POI.

Because several of the genes involved are also cancer-predisposition genes, their identification may have implications for long-term risk assessment and surveillance.

#### 2.6.2. Biallelic *FANCM* Variants Cause Isolated Diminished Ovarian Reserve and POI

We investigated a consanguineous Finnish family in which two sisters, aged 28 and 22 years, presented with isolated unexplained POI and diminished ovarian reserve (DOR) [31]. WES identified a homozygous truncating *FANCM* variant (p.Gln1701Ter). The variant produces a truncated protein lacking the DNA-binding domain. Both parents were heterozygous carriers.

Despite the absence of clinical or hematological signs of FA, cytogenetic analyses performed on lymphoblastoid cells derived from patients demonstrated increased chromosomal instability characterized by elevated frequencies of aberrant metaphases, chromosome breaks, and radial figures after MMC exposure, compared with cells from the mother and healthy controls, strongly supporting dysfunction of the FA pathway (Figure 2A,B; Supplementary Table S2) [31,48]. The heterozygous mother displayed an intermediate phenotype. Neither the patients nor their relatives had clinical manifestations of FA or cancer.

Importantly, *FANCM* is also a cancer susceptibility gene and has been associated with breast, ovarian, head and neck, and colorectal cancers, as well as acute lymphoid leukemia in humans [49]. However, the magnitude of tumor risk specifically in women with POI caused by biallelic *FANCM* variants remains insufficiently defined. These findings indicate that *FANCM* defects may induce sufficient chromosomal instability to impair gametogenesis during fetal life and cause POI, while remaining clinically silent in somatic tissues under basal conditions in the absence of genotoxic agents.

#### 2.6.3. Biallelic Hypomorphic *BRCA2* Variants Cause Isolated POI

BRCA2 (FANCD1) is a key component of the FA pathway and a central mediator of HR during both mitosis and meiosis [1,48]. Heterozygous mutations in *BRCA2* confer susceptibility to breast and ovarian cancers, whereas severe biallelic mutations cause FA.

We reported a 41-year-old patient from a consanguineous Turkish family with POI and primo-secondary amenorrhea, no personal or family history of infertility or cancer, and no clinical or biological features of Fanconi anemia [18]. WES identified the homozygous missense *BRCA2* variant p.Arg2842Cys, located in the DNA-binding domain. Cytogenetic studies of patient-derived cells showed increased chromosomal instability following genotoxic stress induced by MMC, intermediate between that observed in Fanconi anemia cells and normal controls, indicating a partial DNA repair defect. The cytogenetic findings are summarized in Table S2. Although database classifications of this variant have not been fully concordant, functional analyses demonstrated that it is hypomorphic, retaining approximately 33% residual homologous recombination activity [18]. This residual activity may be sufficient to maintain genomic stability in somatic cells under basal conditions but insufficient during fetal meiosis, leading to impaired recombination and infertility.

Importantly, hypomorphic *BRCA2* variants remain associated with a moderate increase in cancer risk. Large case–control studies have shown that missense variants with residual activity between 30–50% are associated with increased breast cancer risk, supporting the need for long-term surveillance [50]. Patients with POI carrying pathogenic or functionally relevant hypomorphic *BRCA2* variants should therefore be referred for specialized oncogenetic evaluation. Surveillance and any risk-reducing intervention should be determined according to the precise genotype, the level of cancer risk established for that variant, age, family history, and current gene-specific oncogenetic recommendations. Presymptomatic testing should also be considered for relatives after appropriate counseling.

Additional cases of isolated POI associated with biallelic *BRCA2* variants were identified in our international cohort [14]. Adult patients with Fanconi anemia carrying biallelic *BRCA2* variants have also been reported to present with POI [19,51].

#### 2.6.4. Biallelic *BRCA1* Variants Reveal a Critical Role in Ovarian Function

We also reported a 14-year-old patient with early-onset severe DOR followed by fluctuating POI [17]. WES identified two heterozygous truncating *BRCA1* variants (p.Ser157Ter and p.Ser264MetfsTer33), both of which had previously been reported in cancer cohorts.

Molecular analyses showed that the exon 11 variant produces a shorter BRCA1 isoform (del11q) retaining partial function, preserving key domains required for DNA repair. No full-length protein was detected.

Cytogenetic studies demonstrated marked chromosomal fragility, with increased spontaneous and MMC-induced chromosomal breaks, consistent with a FA-like cellular phenotype (Figure 2A,C; Table S2). However, the clinical phenotype remained mild, with no malignancy during adolescence and no family history of *BRCA1*-associated cancers.

BRCA1 plays a major role in the repair of DNA double-strand breaks by homologous recombination, notably by facilitating RAD51 loading, coordinating checkpoint activation, and protecting stalled replication forks. In the heterozygous state, pathogenic *BRCA1* variants confer susceptibility to breast and ovarian cancers, whereas biallelic pathogenic variants are a rare cause of FA [48]. The relatively mild clinical phenotype despite truncating variants in a major cancer-predisposition gene may be explained by residual BRCA1 activity.

These findings support a causal role for biallelic *BRCA1* variants in ovarian dysfunction and highlight the critical role of BRCA1 in human ovarian development and fertility.

#### 2.6.5. Other Meiosis and DNA Repair Genes Causing POI

Numerous other genes involved in meiosis and DNA repair have been implicated in POI (Figure 2D and Table S1), further highlighting the central role of genome maintenance in oocyte development. During fetal life, oocytes progress through meiotic prophase I, a highly coordinated process involving chromosome pairing, synapsis, programmed DNA double-strand break, formation and processing, homologous recombination, crossover formation, and chromosome cohesion [1]. Disruption of any of these steps can impair recombination, activate DNA damage checkpoints, and lead to oocyte apoptosis. Accordingly, genes involved in DSB formation and processing, homologous recombination, crossover formation, synaptonemal complex assembly, and chromosome cohesion have been implicated in both isolated and syndromic POI [1,14]. HR is the principal pathway for repairing programmed meiotic DSBs, whereas non-homologous end joining may provide an alternative repair mechanism, particularly during oocyte growth. The Fanconi anemia pathway also cooperates closely with HR to maintain genome stability.

#### 2.6.6. POI-Causing Genes Associated with Cancer Risk in Humans

An increasing number of genes involved in meiosis and DNA repair have been implicated in POI (Figure 2D and Table S1). However, several of these genes, including *BRCA1*, *BRCA2*, and *FANCM*, have also been associated with tumor predisposition in humans (Table 1 [14,17,18,19,20,29,30,31,39,49,50,52,53,54,55,56,57,58,59,60,61,62,63,64,65]). Although most patients are young and asymptomatic at diagnosis, rare tumors have been reported, including an ovarian tumor in *STAG3* deficiency [63] and pilomatricomas in *MCM8* deficiency [58]. Experimental models further support a strong link between defects in these pathways and tumor development [66]. For several genes, however, tumor associations have been established in humans outside the specific context of POI or in a different allelic state; the magnitude of cancer risk in women with POI caused by biallelic variants remains insufficiently defined.

Table 1 summarizes the allelic and clinical context of the reported tumor associations. Importantly, many patients with genetically defined POI are young at ascertainment. The absence of malignancy at diagnosis or in currently reported families may therefore partly reflect age and limited duration of follow-up rather than absence of future risk. Prospective longitudinal studies are required to determine age-dependent, gene-, variant-, and allelic-state-specific cancer risks.

#### 2.6.7. Clinical Implications of Meiosis and DNA Repair Gene Defects in POI

- **Systematic Genetic Testing**

These observations underscore the importance of genetic testing in unexplained POI, as genes involved in meiosis and DNA repair represented the leading molecular category in our international cohort [14]. Even in the absence of overt somatic manifestations, underlying chromosomal instability may be present and can be revealed by cytogenetic studies, particularly in defects affecting the Fanconi anemia pathway.

Identifying this at-risk subgroup is a clinical priority because of the potential for tumor development, particularly in the presence of environmental or treatment-related genotoxic stress, and because it offers opportunities for early surveillance and preventive measures. However, tumor risk depends on the gene, genotype, and allelic state and remains unquantified for many POI-associated defects. Molecular findings should therefore guide gene-specific oncogenetic risk assessment and individualized surveillance rather than uniform cancer screening for all patients.

- **Genetic Counseling and Family Screening**

Pre-test counseling is essential in POI and should address the possibility of identifying variants in cancer-predisposition genes such as *BRCA1* and *BRCA2*. Post-test genetic counseling should be offered to affected patients and, when appropriate, to their relatives, including male relatives and heterozygous carriers. Cascade testing and presymptomatic testing should be considered according to the gene involved, the mode of inheritance, and the associated clinical risks.

- **Long-Term Follow-Up and Prevention**

Assessment of associated comorbidities, including potential tumor predisposition, is important to guide surveillance and enable early diagnosis and management. Long-term multidisciplinary follow-up should include oncogenetic evaluation when appropriate, gene- and genotype-specific surveillance, and genetic counseling. Exposure to avoidable genotoxic agents should be minimized, particularly in patients with demonstrated chromosomal instability.

Cancer screening and risk-reducing interventions should be considered only within a specialized oncogenetic setting and according to established recommendations for the specific gene, variant, allelic state, age, and family history. Gene-specific manifestations should also be considered, including pilomatricomas and colorectal or ovarian tumors reported with *MCM8* deficiency [58,59], and colorectal polyposis and cancer reported with *MCM9* deficiency [60].

- **Specific Clinical Situations: Chromosomal Instability and Growth Hormone Therapy**

In patients with POI associated with short stature, genetic evaluation for DNA-repair or chromosomal-instability disorders should be performed before growth hormone (GH) therapy is considered. In patients with demonstrated chromosomal instability, GH therapy should be avoided or regarded as contraindicated according to specialist recommendations because stimulation of cell proliferation may increase tumor risk [67]. This concern is illustrated by our previous work describing a patient carrying a homozygous *MCM8* variant (p.Arg309Ter) who developed multiple pilomatricomas following GH treatment, thereby defining a novel phenotype combining POI, growth delay, chromosomal instability, and tumor susceptibility [58].

- **Fertility Prognosis and Therapeutic Implications**

Defects affecting meiosis and DNA repair during fetal life are generally associated with severe impairment of ovarian reserve establishment and a poor fertility prognosis, with major implications for counseling and reproductive management. Nevertheless, pregnancies have occasionally been reported. This residual fertility may reflect incomplete penetrance, variable functional consequences of the variants, residual protein activity, or the survival of a limited number of oocytes despite impaired meiotic progression.

### 2.7. Other Genetic Mechanisms in POI: The Central Question of Residual Ovarian Reserve

In our international cohort, 60.4% of patients included in the pathway analysis carried molecular defects affecting follicular growth (35.4%), metabolism and mitochondrial functions (19%), ovarian development (6.1%), immunity, or autophagy [14]. Based on the biological functions of these genes, these molecular categories are consistent with persistence of a residual follicular pool. Preservation of ovarian reserve has also been demonstrated in animal models carrying defects in these pathways [1,14]. However, this 60.4% estimate is derived from pathway assignment and should not be interpreted as direct demonstration of preserved primordial follicles in individual patients.

In POI caused by defects in these pathways, residual ovarian function may initially persist, and detectable AMH levels or spontaneous pregnancies have been reported [14].

However, AMH is only an indirect marker of follicular activity and cannot determine whether the primordial or primary follicle pool is depleted or preserved.

Human histological data support the biological basis of this distinction. In Massin et al., ovarian biopsies from 67 women with premature ovarian insufficiency identified follicles ranging from primordial to antral stages in 29 patients (43.3%), whereas 38 patients had no detectable follicles [68]. These patients were drawn from a cohort of 166 women aged 16–39 years, with a median age at POI diagnosis of 28 years. These findings provide direct evidence that a substantial subgroup of women with POI retains a follicular pool. However, the interval between POI onset or diagnosis and ovarian biopsy was not systematically reported; therefore, absence of follicles at biopsy does not establish that a residual pool had never been present earlier in the course of the disease.

The persistence of residual ovarian activity is also likely to be time-dependent. In the longitudinal study of Bidet et al., spontaneous resumption of ovarian function occurred in 24% of women with idiopathic POI, with 88% of these events occurring within the first year after diagnosis [69]. Thus, the clinical demonstration of residual ovarian activity depends not only on the underlying cause but also on the time course of POI.

Prospective studies integrating genetic diagnosis with longitudinal assessment of ovarian activity and spontaneous pregnancies will be important to determine whether specific genetic causes are associated with the probability and duration of residual ovarian function.

Together, these observations support a mechanistic distinction between POI caused by impaired establishment or accelerated depletion of the ovarian reserve and forms in which a residual follicular pool may persist. These findings have important translational implications. Early genetic diagnosis may identify patients and at-risk relatives in whom persistence of a residual follicular pool is plausible, thereby revealing a potentially limited window of opportunity for fertility preservation before further follicular depletion occurs. They also provide a biological rationale for the development of mechanism-based targeted therapies aimed at promoting follicular activation, growth, or maturation [70,71,72]. These clinical implications are discussed in Section 2.9.

The following examples illustrate how complete loss of FSHR or BMP15 function can cause severe ovarian dysfunction despite the initial persistence of a residual pool of small follicles.

#### 2.7.1. Increased Small-Follicle Density in POI Associated with Complete Loss of FSHR Function

We reported a patient carrying a homozygous inactivating *FSHR* variant (p.Pro519Thr), resulting in complete loss of receptor function [73]. The patient presented with POI, delayed puberty, and primary amenorrhea. Ultrasonography showed small ovaries without visible follicles, and no response was observed after administration of high doses of recombinant FSH *in vivo*.

However, histological analysis revealed a block in follicular growth after the primary stage, demonstrating that FSH is essential for subsequent follicular maturation in humans (Figure 3A–C). Moreover, the density of primordial and primary follicles was remarkably high, approximately 38-fold greater than in age-matched controls, and comparable to that observed in prepubertal ovaries (Figure 3D,E).

These findings indicate that ovarian reserve is preserved in patients with POI caused by complete *FSHR* inactivation. This condition should therefore be considered primarily a disorder of follicular growth rather than premature depletion of the ovarian reserve. This distinction has important implications for fertility counseling.

From a therapeutic perspective, the presence of numerous small follicles suggests that future interventions aimed at promoting follicular growth and maturation may be possible.

Previously published studies from our team also described patients with partial loss-of-function *FSHR* variants retaining residual receptor activity [74]. In these cases, follicular development may progress to the antral stage, allowing ultrasound visualization. High-dose FSH stimulation combined with *in vitro* maturation has enabled pregnancies in some patients [75], illustrating the clinical importance of genotype–phenotype correlations.

#### 2.7.2. *BMP15* “Knockout-like” Effect in Familial POI with a Residual Ovarian Reserve

BMP15, an oocyte-derived growth factor belonging to the transforming growth factor-β (TGF-β) superfamily, acts in cooperation with GDF9 to regulate granulosa-cell proliferation and follicular development [76]. We identified compound heterozygous truncating *BMP15* variants in two sisters, resulting in a human knockout-like phenotype [77].

Both patients presented with POI characterized by primary or primo-secondary amenorrhea, and ovarian ultrasonography showed no visible follicles. However, AMH levels were initially low but detectable, indicating residual follicular activity despite the absence of ultrasonographically detectable follicles. Five years later, AMH had become undetectable in both sisters, and one sister had undergone oocyte donation.

These findings support the concept that complete loss of BMP15 function may initially be compatible with persistence of a residual follicular pool, followed by progressive depletion. Early genetic diagnosis may therefore reveal a limited window of opportunity during which fertility-preservation strategies could still be considered, before residual follicular activity is lost.

Unlike complete *FSHR* inactivation, for which preservation of the small-follicle pool was demonstrated histologically, knockout-like *BMP15* deficiency provides indirect evidence of residual follicular activity based on detectable AMH and its subsequent decline.

### 2.8. Genetic Reclassification of POI: Translational Implications for Comorbidities and Fertility Prognosis

#### 2.8.1. Syndromic POI Unmasked by Genetic Diagnosis

In our international cohort, 20% of syndromic POI cases initially appeared isolated and were subsequently reclassified through genetic testing [14]. Comprehensive clinical evaluation of patients and their families is therefore essential to identify associated features that may initially be overlooked, depending on the causal gene. These may include neurological, cardiological, sensory, metabolic, endocrine, or dysmorphic manifestations, ovarioleukodystrophy, and susceptibility to tumors or cancers, particularly in the context of meiosis and DNA repair defects [1].

Management of these patients requires a multidisciplinary approach tailored to the organs affected. Consistently, a population-based Finnish registry study including more than 5000 women with POI reported that 15.9% had at least one additional congenital disorder [78]. In our previously published international cohort, 44.8% of patients had, or were at risk of developing, associated comorbidities requiring multidisciplinary assessment, and pathogenic variants in genes causing syndromic POI were identified in 8.5% of cases [14].

#### 2.8.2. Example of Genetic Reclassification: A Metabolic Cause with a Favorable Fertility Prognosis

In our international cohort, we identified a patient with POI, secondary amenorrhea, small ovaries on ultrasound, and undetectable AMH levels [14]. NGS identified a homozygous pathogenic *GALT* variant (c.563A>G; p.Gln188Arg), the most frequent variant responsible for galactosemia [79], a severe metabolic disorder usually diagnosed in the neonatal period.

Clinical reassessment after genetic diagnosis revealed a history of early-onset cataracts requiring surgery in childhood and learning difficulties, features consistent with adult survivors of galactosemia but not initially reported. This case illustrates how genetic diagnosis can reclassify apparently isolated POI as part of a syndromic disorder.

It is important to note that galactosemia is associated with a relatively favorable fertility prognosis compared to other causes of POI. The spontaneous pregnancy rate is estimated at 48% at 2 years [80], compared to approximately 90% in the general population and ~5% in other forms of POI [81,82].

This diagnosis has major implications for genetic counseling and management. Patients should be informed of the possibility of spontaneous pregnancy, which may lead to reconsideration of assisted reproductive strategies, as illustrated by the patient who discontinued an oocyte-donation program.

### 2.9. Clinical Implications of Non-DNA Repair Gene Defects in POI

- **Systematic Genetic Testing and Molecular Stratification**

Molecular stratification may help distinguish disorders of follicular growth or function from conditions characterized by severe depletion of the ovarian reserve. In non-DNA repair forms of POI, identification of the causal gene may provide clinically relevant information on the underlying ovarian mechanism and on whether a residual follicular pool may persist. In our international cohort, 60.4% of patients included in the pathway analysis had defects in biological pathways compatible with residual ovarian reserve, including follicular growth, metabolic and mitochondrial functions, ovarian development, immunity, and autophagy [14]. This 60.4% estimate is based on pathway assignment and does not directly demonstrate preservation of primordial follicles in individual patients. Nevertheless, molecular stratification may identify forms of POI in which residual ovarian function is more likely to persist, thereby defining a clinically important and potentially time-limited window of opportunity for fertility preservation. The likelihood and duration of residual ovarian function are expected to vary according to the underlying gene, variant, and disease duration and require prospective evaluation.

Genetic diagnosis therefore complements clinical and biochemical assessment of ovarian function, because AMH levels and ovarian ultrasonography cannot reliably determine whether primordial or primary follicles remain, especially when follicular development is arrested before the antral stage.

- **Genetic Counseling and Family Screening**

Post-test genetic counseling should explain the causal mechanism, mode of inheritance, expected ovarian phenotype, and limitations of individual fertility prediction. When appropriate, cascade testing should be proposed to female relatives at risk of DOR or POI.

Identification of an at-risk relative before overt ovarian insufficiency may allow earlier assessment of ovarian function and timely discussion of fertility-preservation strategies. Male relatives should also be considered when the causal gene is associated with male infertility or broader reproductive phenotypes.

- **Gene-Specific Assessment of Associated Comorbidities and Multidisciplinary Follow-Up**

Non-DNA repair POI may occur as an isolated condition or as the reproductive manifestation of a broader genetic syndrome. Molecular diagnosis can reveal associated metabolic, mitochondrial, neurological, cardiological, endocrine, sensory, developmental, or immune manifestations that were not recognized at the time of POI diagnosis. Clinical reassessment should therefore be guided by the causal gene and include targeted evaluation of potentially associated manifestations. Multidisciplinary follow-up may be required, particularly when the genetic diagnosis indicates a syndromic form of POI. The identification of a metabolic cause may also substantially modify reproductive counseling, as illustrated by POI caused by biallelic pathogenic variants in *GALT*, in which spontaneous pregnancies occur more frequently than in many other genetic forms of POI [80].

- **Fertility Prognosis and Early Fertility Preservation**

In the overall POI population, intermittent resumption of ovarian activity occurs in approximately 23% of patients, and reported spontaneous pregnancy rates range from 2.2% to 14.2% [81,82]. In non-DNA repair forms of POI, the underlying molecular defect may help identify patients in whom an initial follicular pool is more likely to persist. However, pathway-based stratification cannot currently predict individual reproductive outcomes with certainty. For some defects, such as complete *FSHR* inactivation or knockout-like *BMP15* deficiency, preservation of the ovarian reserve is strongly supported by the underlying mechanism and available clinical evidence. For other genes, the extent and duration of residual ovarian function may vary and require further genotype-specific evaluation.

Fertility counseling should therefore integrate the causal gene and molecular mechanism with the patient’s age, menstrual history, interval since POI diagnosis, AMH levels, ultrasonographic findings, and any evidence of intermittent ovarian activity. Early referral to a reproductive medicine specialist should be considered for affected patients and genetically at-risk relatives when persistence of a residual ovarian reserve is plausible. Oocyte or ovarian-tissue cryopreservation should be discussed on an individualized basis and, whenever possible, before further follicular depletion occurs. Ovarian-tissue cryopreservation should not be proposed when available evidence indicates that the follicular pool is already severely depleted.

- **Perspectives for Mechanism-Based Targeted Therapies**

Molecular stratification may improve the selection of patients for emerging therapies aimed at exploiting or preserving a residual follicular pool. Patients with defects affecting follicular activation, growth, maturation, metabolism, or mitochondrial function may represent more biologically appropriate candidates for mechanism-based interventions than unselected populations of patients with POI. Ovarian stromal fibrosis may represent an additional therapeutic target in selected patients, irrespective of the primary genetic mechanism.

Among the approaches currently under investigation, *in vitro* activation of dormant primordial follicles aims to promote follicular recruitment through modulation of the Hippo and PI3K-AKT-FOXO3 pathways [70]. When antral follicles are present, *in vitro* maturation of retrieved immature oocytes may also be considered [75]. In *FSHR*-related POI, therapeutic responsiveness depends on residual receptor activity, illustrating how genotype–phenotype correlations may guide individualized treatment strategies [73,74,75]. Similar approaches may be relevant in selected patients with partial defects in other follicular-growth genes, although supporting clinical evidence remains limited. Patients with metabolic or mitochondrial defects may also be candidates for future targeted therapies. Finerenone has recently been reported to reduce ovarian stromal fibrosis and promote follicular development in experimental models, with preliminary clinical observations suggesting recovery of antral follicle development and retrieval of mature oocytes in some patients with POI [71]. These findings require independent replication and evaluation in controlled clinical studies. Finally, resveratrol has been reported to enhance DNA-damage repair in experimental models of POI through multi-target modulation of the homologous recombination pathway [72].

Prospective genotype-specific studies are now required to determine whether molecular classification can reliably predict spontaneous ovarian activity, response to stimulation, success of fertility preservation, or eligibility for emerging mechanism-based approaches targeting the identified causal defect in patients with residual follicular activity.

### 2.10. Recent International Guidelines and Translational Integration of POI Genetics

In France, work from our team led to the inclusion of POI in the French Genomic Medicine Plan (PFMG) in 2020, enabling access to WGS for patients with unexplained POI through two dedicated national sequencing platforms [83]. This initiative aims to promote equitable access to genomic medicine and support innovative approaches to diagnosis and clinical management.

In late 2024, the European Society of Human Reproduction and Embryology (ESHRE), the International Menopause Society (IMS), the American Society for Reproductive Medicine (ASRM), and the Centre for Research Excellence in Women’s Health in Reproductive Life (CRE WHiRL), jointly published updated international guidelines for the diagnosis and management of POI. Drawing largely on evidence from large cohort studies using NGS [14,16], these guidelines formally integrated broader genetic testing, including NGS where available, into the etiological evaluation and management of POI [2]. A clinical decision-making algorithm based on these recommendations is presented in Figure 4.

Current recommendations emphasize that the implications of genetic testing should be discussed with patients before testing and that patients should be offered genetic counseling when clinically indicated. Chromosomal analysis is recommended for all women with unexplained POI. In patients with a normal karyotype, additional genetic testing should be offered after appropriate pre-test information and informed consent, including parallel *FMR1* analysis and NGS performed on the same patient sample to identify monogenic causes of POI.

The principal translational challenge is now to ensure equitable access to validated genetic testing, harmonized variant interpretation, appropriate genetic counseling, periodic reanalysis of negative results, and integration of gene-specific findings into reproductive and long-term care. National genomic programs can support this implementation by providing standardized testing pathways, expert variant interpretation, and coordinated access to genetics, reproductive medicine, and multidisciplinary care.

Post-test genetic counseling should address the clinical significance of the identified variant for the patient and the risk of transmission to offspring, including following spontaneous conception. Counseling should be tailored to the mode of inheritance and include partner testing when relevant, cascade testing of relatives, and discussion of available reproductive options. Particular attention is required when variants are identified in genes associated with repeat-expansion disorders, cancer predisposition, or syndromic forms of POI because of their implications for patients, offspring, and other relatives. For *FMR1* premutation carriers, counseling should specifically address the risk of repeat expansion and fragile X syndrome in offspring. Variants in cancer-predisposition genes may require oncogenetic evaluation and gene-specific surveillance, whereas syndromic forms may warrant multidisciplinary assessment and anticipatory management of associated comorbidities.

Using this diagnostic strategy, our cohort study established a molecular diagnosis in approximately one-third of patients with unexplained POI [14]. This diagnostic yield is expected to increase as additional causative genes are identified and validated.

## 3. Conclusions and Perspectives: From Genetic Diagnosis to Precision Medicine

Genetic advances have transformed POI into a clinically actionable condition. Genomic studies have revealed a highly heterogeneous group of disorders involving meiosis and DNA repair, follicular development and signaling, mitochondrial and metabolic pathways, and multisystemic mechanisms. In patients with unexplained POI, genetic testing can now establish a molecular diagnosis in a substantial proportion of cases. Chromosomal analysis and *FMR1* premutation testing are established components of the evaluation of unexplained POI, while broader NGS is now recommended in recent international guidelines where available and is increasingly used to identify monogenic causes. The clinical implications of an identified genetic defect depend on the specific gene and variant.

Identification of the causal genetic defect refines diagnosis, clarifies the mode of inheritance, may inform reproductive prognosis, and supports personalized management. It enables the detection of subtle or initially absent syndromic manifestations and, for certain genotypes, the identification of a predisposition to tumors or other long-term comorbidities, while guiding cascade screening and genetic counseling for relatives.

Variants affecting genome maintenance require particular attention because ovarian dysfunction may precede somatic manifestations of chromosomal instability or cancer predisposition. Oncogenetic assessment and surveillance should therefore remain gene-, variant-, and allelic-state specific.

Molecular stratification may also distinguish defects associated with impaired establishment or accelerated depletion of the ovarian reserve from those compatible with persistence of a residual follicular pool. In non-DNA repair forms of POI, early molecular diagnosis may identify selected patients and at-risk relatives with a potentially limited window of opportunity for fertility preservation or future targeted interventions. However, the likelihood and duration of residual ovarian function remain gene- and variant-dependent and require prospective evaluation.

Future research should prioritize functional validation of candidate variants using human-relevant models and prospective gene-specific studies. For defects affecting meiosis and DNA repair, the major priority is to define the magnitude and spectrum of cancer risk according to the gene, variant, and allelic state.

For non-DNA repair forms of POI, prospective studies should determine the likelihood and duration of residual ovarian function, spontaneous or assisted pregnancy, and response to fertility-preservation or targeted therapeutic strategies.

By linking molecular diagnosis to individualized reproductive management and long-term care, POI represents a compelling model of precision medicine in female infertility and reproductive endocrinology.

## Figures and Tables

**Figure 1:**
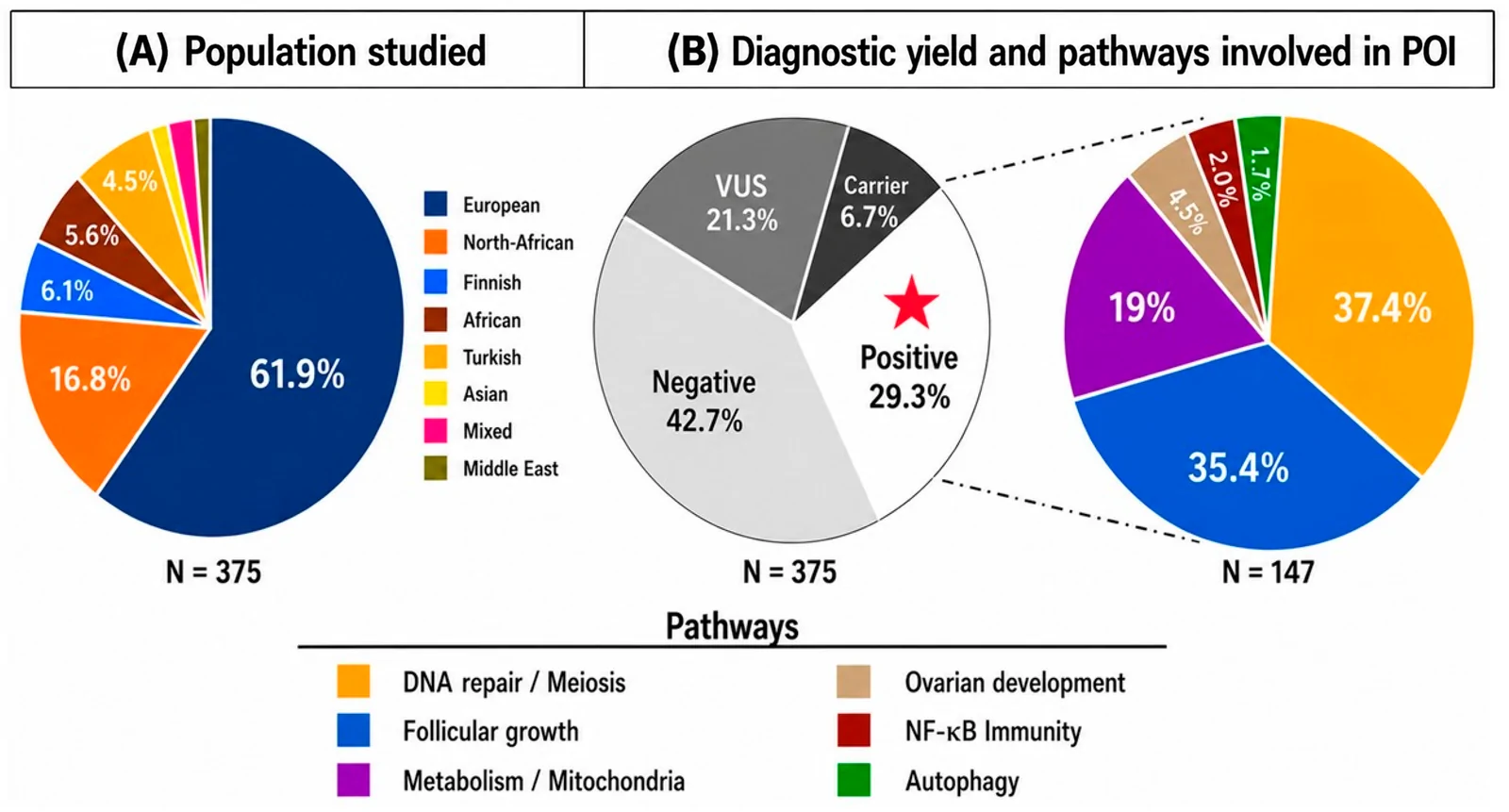
**Genetic analysis of a large international cohort of patients with premature ovarian insufficiency**. Patients were analyzed using a custom 88-gene next-generation sequencing panel or whole-exome sequencing. (**A**) Study population (*N* = 375), showing the geographic origin of patients with unexplained premature ovarian insufficiency (POI). (**B**) Diagnostic yield and biological pathways. **Left**, ACMG-based variant classification in the complete cohort (*N* = 375). “Positive” (red star) denotes patients carrying pathogenic (P) or likely pathogenic (LP) variants compatible with a molecular diagnosis of POI (110/375; 29.3%). “VUS” denotes variants of uncertain significance not retained for molecular diagnosis. “Carrier” denotes patients carrying a single heterozygous P/LP variant in a gene associated with autosomal recessive POI; these variants were not included in the diagnostic yield. **Right**, distribution of the 147 P/LP variants identified in the cohort across the main biological pathways. When two P/LP variants were identified in the same gene in a given patient, the gene and corresponding pathway were counted once. Heterozygous P/LP variants classified as carrier status in autosomal recessive POI genes were included in the pathway analysis but not in the diagnostic yield. The main pathways were meiosis/DNA repair and mitosis (37.4%), follicular growth (35.4%), mitochondrial and metabolic functions (19.0%), ovarian development (6.1%), NF-κB signaling (1.4%), and autophagy (0.7%). Adapted from Heddar et al. [14], with permission from Elsevier.

**Figure 2:**
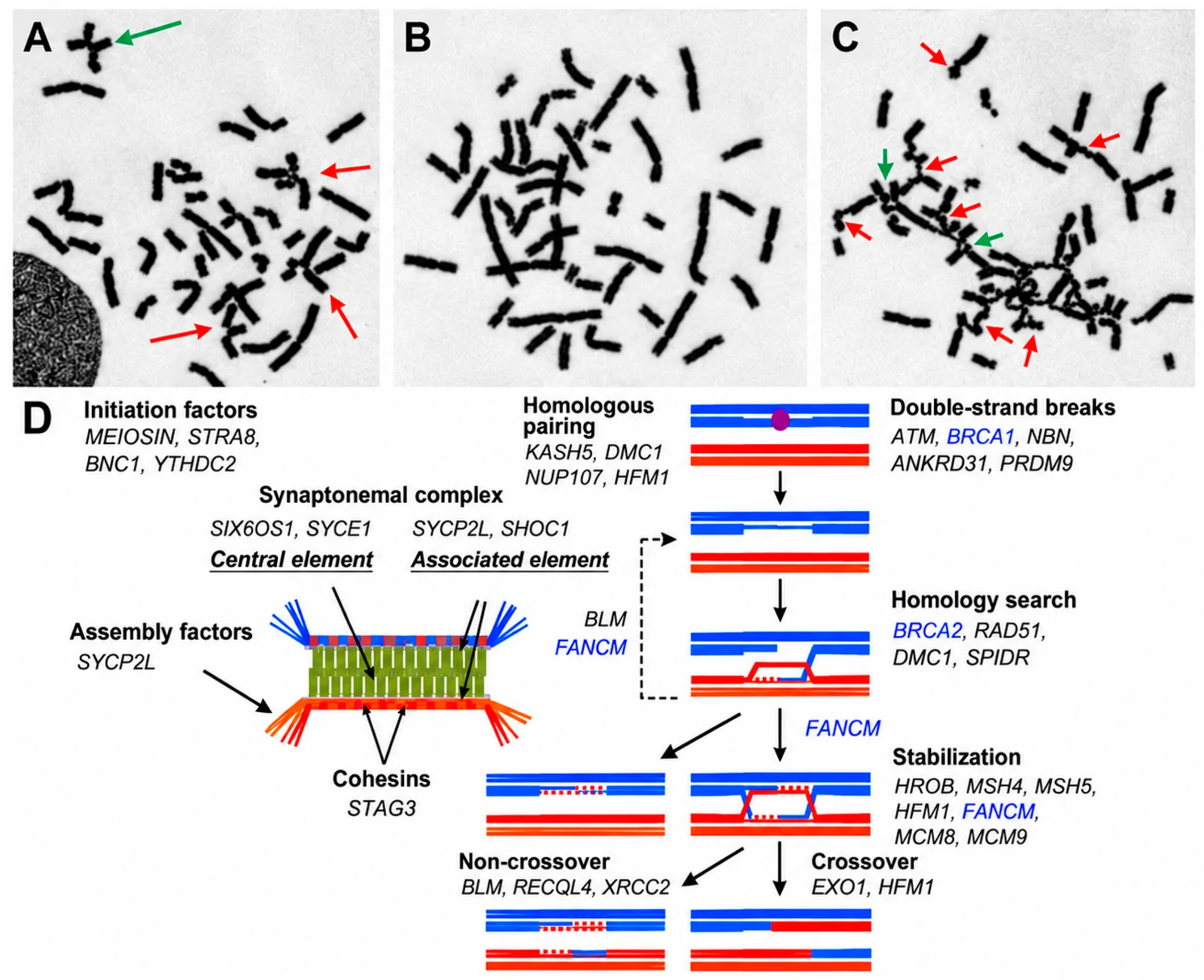
**Chromosomal instability and meiotic DNA-repair pathways implicated in premature ovarian insufficiency.** Top panels: (**A**–**C**) Cytogenetic analysis of lymphoblastoid cells following exposure to mitomycin C (MMC). Representative metaphases show increased chromosomal instability in cells from patients carrying biallelic *FANCM* (**A**) or *BRCA1* (**C**) variants compared with control cells (CTRL) (**B**), including chromosome breaks (red arrows) and radial figures (green arrows). Quantitative cytogenetic findings for *FANCM*-, *BRCA1*-, and *BRCA2*-associated samples are summarized in Table S2. The MMC concentrations used in the original studies differed: 5 µM for *FANCM* and 150 nM for *BRCA1*. The *FANCM* image was adapted from Fouquet et al. [31] under the terms of the Creative Commons Attribution (CC BY) license, and the *BRCA1* image was adapted from Helbling-Leclerc et al. [17] under the terms of the Creative Commons Attribution (CC BY) license. Panel (**D**): Schematic representation of meiotic DNA-repair pathways implicated in POI. Defects in double-strand break formation and repair, homologous chromosome pairing and recombination, synaptonemal-complex assembly, crossover formation, and chromosome cohesion impair meiotic progression and promote oocyte depletion. Selected genes implicated in human POI are indicated at the corresponding steps, with *FANCM*, *BRCA1*, and *BRCA2* highlighted in blue. Adapted from Huhtaniemi et al. [1], with permission from Elsevier.

**Figure 3:**
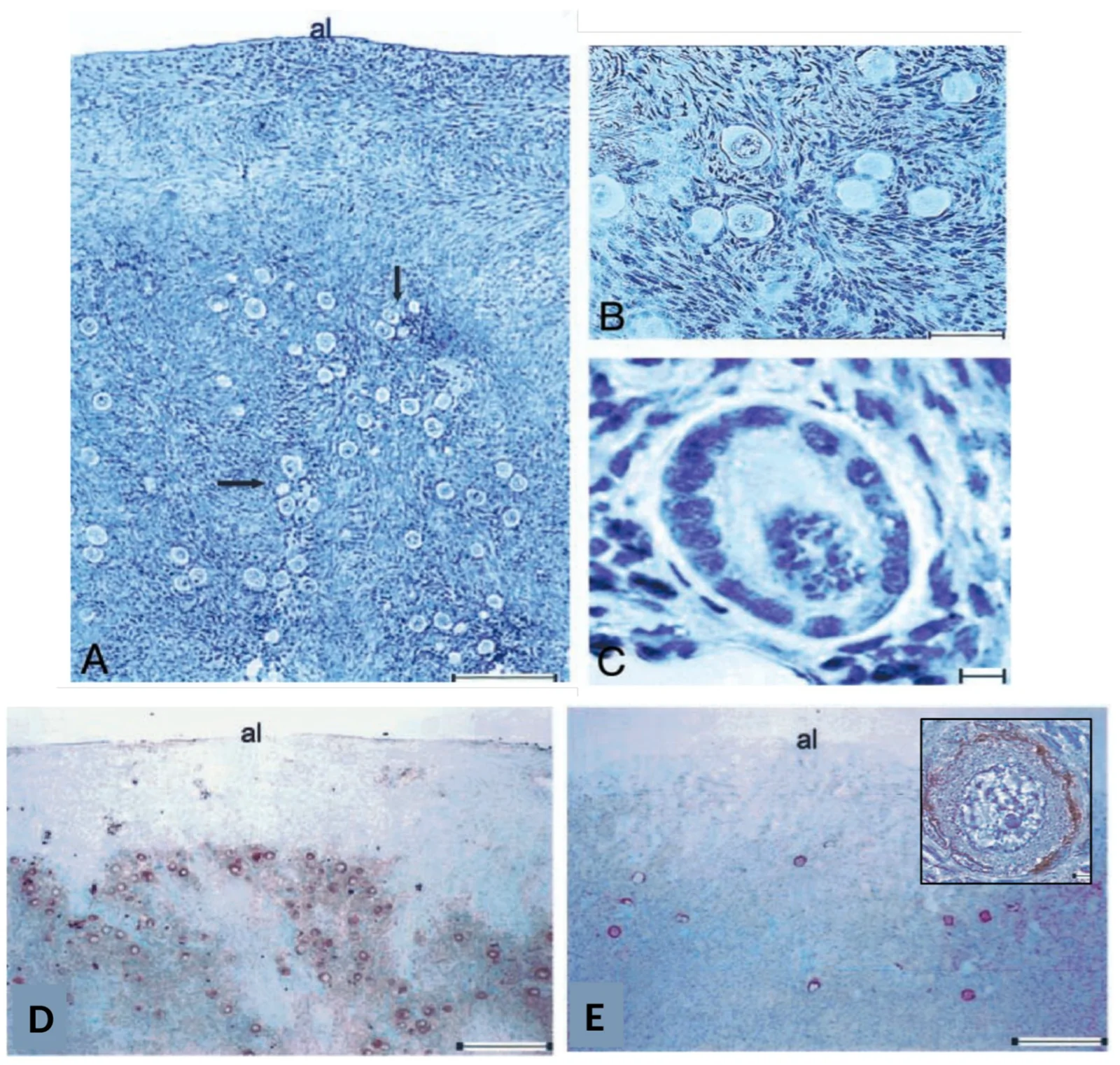
**Ovarian histology and immunohistochemical analysis in a patient with POI and complete inactivation of the follicle-stimulating hormone receptor.** (**A**) Ovarian cortex showing numerous primordial, intermediary, and primary follicles beneath the albuginea (al; arrows). (**B**) Higher-magnification view showing clusters of small follicles within the ovarian stroma. (**C**) Representative primary follicle composed of a central oocyte surrounded by a single layer of cuboidal granulosa cells. No follicles beyond the primary stage are observed, indicating arrest of follicular maturation. (**D**,**E**) Anti-c-Kit immunohistochemistry. (**D**) Patient ovarian tissue showing a marked increase in small follicles, predominantly at primordial and intermediary stages. (**E**) Age-matched control ovarian tissue showing substantially lower follicular density. ((**E**), inset) Representative primary follicle showing c-Kit expression at the oocyte membrane. Follicular density was markedly increased in the patient compared with an age-matched control. Scale bars: (**A**,**D**,**E**), 200 µm; (**B**), 50 µm; (**C**), 10 µm. Abbreviations: al, tunica albuginea. Adapted from Meduri et al. [73], with permission from Oxford University Press.

**Figure 4:**
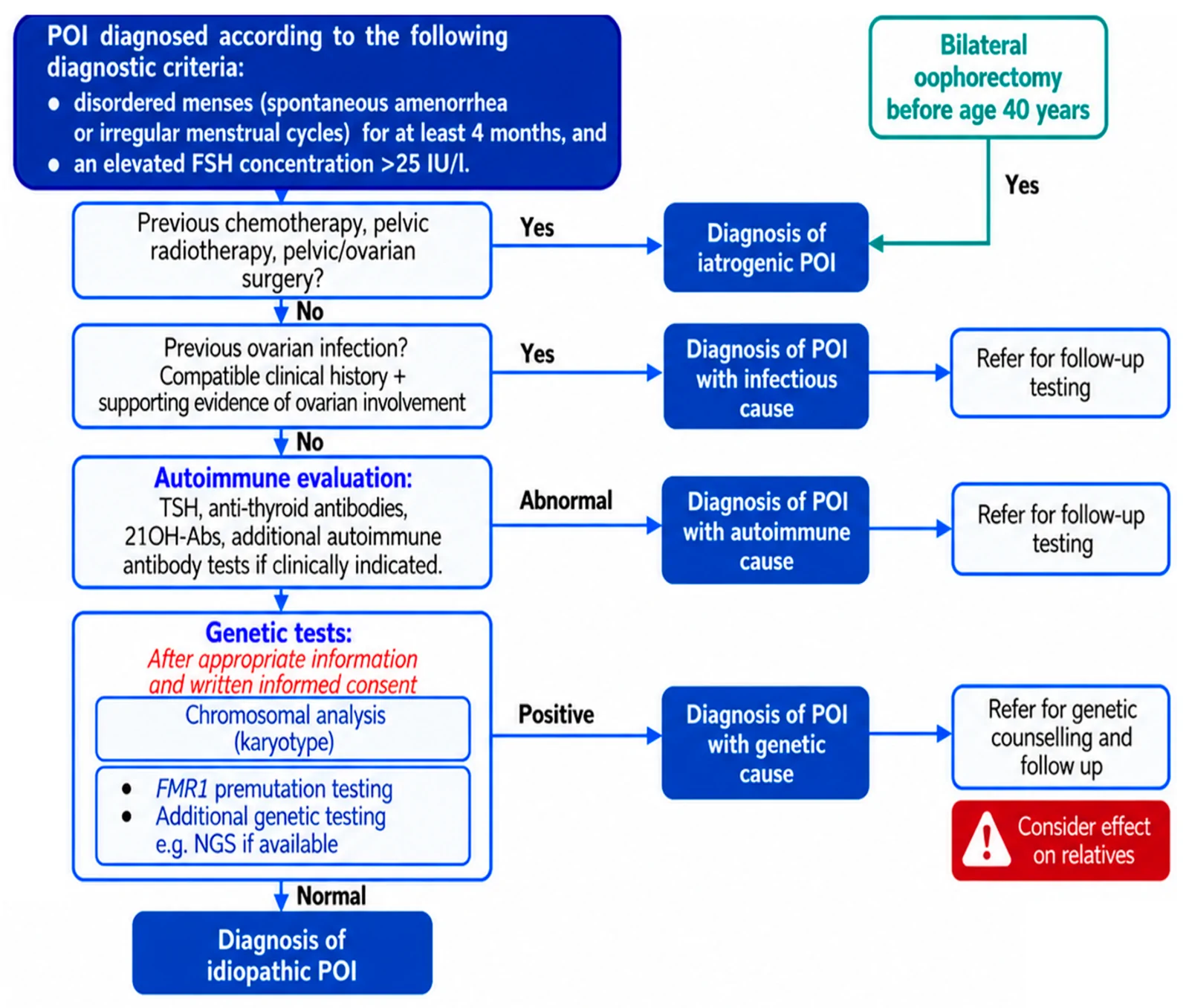
**Etiological evaluation of premature ovarian insufficiency (POI).** Summary of recommendations for the diagnosis and etiological evaluation of POI. POI is diagnosed in women younger than 40 years on the basis of disordered menstrual cycles (amenorrhea or irregular menses for at least 4 months) and an elevated follicle-stimulating hormone concentration (>25 IU/L). Iatrogenic causes are identified from treatment and surgical history. Infectious POI requires a compatible clinical history together with supporting evidence of ovarian involvement; history alone is insufficient. Autoimmune evaluation includes TSH, anti-thyroid antibodies, and 21-hydroxylase antibodies (21OH-Abs), with additional autoimmune testing when clinically indicated. An abnormal TSH indicates thyroid dysfunction but does not by itself establish autoimmune POI, whereas positive 21OH-Abs support an autoimmune etiology in the appropriate clinical context. Genetic evaluation is not dependent on a normal autoimmune evaluation. It includes chromosomal analysis and, when the karyotype is normal, *FMR1* premutation testing, together with parallel additional testing, i.e., NGS where available. If no specific cause is identified, POI is classified as idiopathic. Adapted from Panay et al. [2] under the terms of the Creative Commons Attribution (CC BY) license.

**Table 1:** Selected human meiosis and DNA repair genes implicated in POI and linked to tumors or cancers in humans.

Gene Name	Inheritance in POI	Associated Clinical Features	Human Tumor Evidence and Reported Tumors/Cancers	Tumor Specifically Reported in POI Patients	Male Infertility	References
*ATM*	AR	Ataxia-telangiectasia	Germline predisposition—monoallelic; biallelic, syndromic (ataxia-telangiectasia). Leukemia/lymphoma, breast and other solid tumors	No	NR	[14,52]
*BLM* (*RECQL3*)	AR	Bloom syndrome	Germline predisposition—biallelic, syndromic (Bloom syndrome). Broad cancer spectrum.	No	Yes	[14,53]
*BRCA1* (*FANCS*)	AR	DOR; Fanconi anemia-like phenotype	Germline predisposition—monoallelic; biallelic, FA/FA-like. Breast, ovarian, pancreatic cancers.	No	NR	[17,54,55]
*BRCA2 (FANCD1)*	AR	Isolated POI or Fanconi anemia	Germline predisposition—monoallelic; biallelic, syndromic (FA). Breast, ovarian, prostate, pancreatic cancers; leukemia.	No	Yes	[18,19,50]
*BRIP1* (*FANCJ*)	AR	Fanconi anemia	Germline predisposition—monoallelic; biallelic, syndromic (FA). Ovarian cancer.	No	NR	[20,56]
*FANCA*	AR	Fanconi anemia	Germline predisposition—biallelic, syndromic (FA). Hematologic malignancies; SCC.	No	Yes	[29,57]
*FANCI*	AR	Fanconi anemia	Germline predisposition—biallelic, syndromic (FA). Hematologic malignancies; SCC.	No	Yes	[30,57]
*FANCM*	AR	Chromosomal instability/ Fanconi-pathway defect	Germline tumor association—monoallelic; biallelic, FA-pathway context. Breast, ovarian, colorectal cancers; leukemia.	No	Yes	[31,49]
*MCM8*	AR	Growth retardation; hypothyroidism	Tumor association—biallelic; predisposition not established. Pilomatricomas; colorectal/ovarian tumors reported.	Yes (*n* = 1; multiple pilomatricomas)	Yes	[14,58,59]
*MCM9*	AR	Short stature; hypothyroidism	Germline predisposition—mainly biallelic. Colorectal polyposis/cancer; other early-onset tumors.	Yes (reported POI cases; colorectal, cervical or germ-cell tumors)	Yes	[14,59,60]
*NBN*	AR	Nijmegen breakage syndrome	Germline predisposition—biallelic, syndromic (Nijmegen breakage syndrome). Hematologic malignancies; breast and brain tumors.	No	Yes	[14,61]
*PSMC3IP*	AR	Not reported	Germline association; somatic second hits reported. Breast and ovarian tumors.	No	Yes	[14]
*RAD51* (*FANCR*)	AD/rare AR	Fanconi-like phenotype	Germline/FA-spectrum association; allele dependent. Breast and FA-spectrum malignancies reported.	No	Yes	[39]
*RECQL4*	AR	Rothmund–Thomson syndrome	Germline predisposition—biallelic, syndromic (Rothmund-Thomson syndrome). Osteosarcoma and related cancers.	No	NR	[14,62]
*STAG3*	AR	Not reported	Tumor association—biallelic; predisposition not established. Ovarian tumor.	Yes (*n* = 1; ovarian tumor)	Yes	[63]
*WRN*	AR	Werner syndrome	Germline predisposition—biallelic, syndromic (Werner syndrome). Broad cancer spectrum.	No	Yes	[14,64]
*XRCC2* (*FANCU*)	AR	Fanconi-like phenotype	Germline association—monoallelic; biallelic, FA-like context. Hematologic and breast tumors reported.	No	Yes	[39,65]

Human tumor evidence is presented according to its allelic and clinical context, including monoallelic or biallelic status and syndromic context where relevant. “Tumor specifically reported in POI patients” indicates direct observation of a tumor in a patient with POI carrying the relevant genotype; when available, the number and tumor type are indicated. “No” indicates that no tumor has been specifically reported in patients with POI carrying the relevant genotype, although a tumor association may have been reported in another allelic or syndromic context. An isolated tumor report should not be interpreted as evidence of increased cancer risk in all patients with POI. Because many genetically characterized patients with POI are young and longitudinal follow-up remains limited, absence of malignancy at ascertainment does not exclude a later age-dependent risk. Longitudinal, genotype-specific studies are required to determine such risks. Somatic tumor alterations alone are not considered evidence of inherited cancer predisposition. Male infertility: “Yes” indicates that a human male infertility or reproductive phenotype has been reported in association with pathogenic variants in the same gene; “NR” indicates that such a phenotype was not reported in the sources used for the table. AD, autosomal dominant; AR, autosomal recessive; DOR, diminished ovarian reserve; FA, Fanconi anemia; POI, premature ovarian insufficiency; SCC, squamous cell carcinoma.

## Data Availability

No new data were created or analyzed in this study. Data sharing is not applicable to this article.

## Supplementary Materials

The following supporting information can be downloaded at https://www.mdpi.com/article/10.3390/ijms27198662/s1.
