# Supplementary material for: Genetics of Premature Ovarian Insufficiency: From Molecular Architecture to Precision Medicine
Source: Int J Mol Sci. 2026 Sep 28;27(19):8662. doi: 10.3390/ijms27198662 (PMC13665432; doi:10.3390/ijms27198662)
Supplement: Supplementary file 1 [file ijms-27-08662-s001.zip › Sousa_POI_Genetics_IJMS_Table_S2.pdf]

Supplementary Table S2. Mitomycin C–induced chromosomal instability in patients with POI carrying pathogenic variants in *FANCM, BRCA1,* and *BRCA2*.

Cytogenetic analysis of patient-derived lymphoblastoid cell lines showing chromosomal fragility before and after exposure to mitomycin C (MMC). Aberrant metaphases, chromosome breaks per metaphase, and radial figures were quantified in control cells, a heterozygous FANCM carrier (the patient's mother), and patients carrying biallelic pathogenic variants in *FANCM*, *BRCA2*, or *BRCA1*.

Data for the *FANCM* samples were derived from Fouquet et al. [31], and data for the *BRCA2* sample were derived from Caburet et al. [18]. Permission to reproduce these data has been requested. Data for the *BRCA1* sample were derived from Helbling-Leclerc et al. [17] and are reproduced under the terms of the Creative Commons Attribution (CC BY) license.

MMC concentrations differed among experiments: 5 µM for the FANCM samples, 1000 nM for the BRCA2 sample, and 150 nM for the *BRCA1* sample. Because these data were obtained in independent studies under different experimental conditions, direct quantitative comparisons between genotypes should be interpreted with caution.

Abbreviations: MMC, mitomycin C; WT, wild type.

(continued on next page)

		Aberrant Metaphases		Chromosome breaks per metaphase		Radial figures	
**Individual**	**Genotype**	**Untreated**	**MMC**	**Untreated**	**MMC**	**Untreated**	**MMC**
**Control**	WT	0/50 (0%)	1/50 (2%)	0.00	0.02	0/50 (0%)	0/50 (0%)
**Mother of the FANCM patient**	Heterozygous	7/50 (14%)	14/50 (28%)	0.22	0.40	0/50 (0%)	1/50 (2%)
**FANCM patient**	Homozygous	31/50 (62%)	37/50 (74%)	1.12	2.48	0/50 (0%)	7/50 (14%)
**BRCA2 patient**	Homozygous	1/50 (2%)	11/25 (44%)	0.02	1.04	0/50 (0%)	4/25 (16%)
**BRCA1 patient**	Compound heterozygous	4/50 (8%)	48/50 (96%)	0.08	8.74	0/50 (0%)	Numerous
